# Changes in Oral Microbial Ecology of C57BL/6 Mice at Different Ages Associated with Sampling Methodology

**DOI:** 10.3390/microorganisms7090283

**Published:** 2019-08-22

**Authors:** Angélica Hernández-Arriaga, Anja Baumann, Otto W. Witte, Christiane Frahm, Ina Bergheim, Amélia Camarinha-Silva

**Affiliations:** 1 Institute of Animal Science, University of Hohenheim, 70599 Stuttgart, Germany; 2 Department of Nutritional Sciences, Molecular Nutritional Science, University Vienna, 1090 Vienna, Austria; 3 Hans Berger Department of Neurology, Jena University Hospital, 07747 Jena, Germany

**Keywords:** oral microbiota, oral swab, oral tissue biopsy, microbial ecology, aging, sampling methodologies

## Abstract

The mouth is an important niche for bacterial colonization. Previous research used mouth microbiota to predict diseases like colon cancer and inflammatory bowel disease (IBD). It is still unclear how the sampling methodology influences microbial characterization. Our aim was to determine if the sampling methods, e.g., cotton swab or tissue biopsy, and the age influence the oral microbial composition of mice. Microbial DNA was extracted using a commercial kit and characterized targeting the 16s rRNA gene from mouth swabs and tissue biopsies from 2 and 15 months old C57BL/6 male mice kept in the same SPF facility. Our results show statistical different microbial community of the different ages, type of sampling, and the two fixed factors age x type of sample (*p*-value < 0.05). At the genus level, we identified that the genera *Actinobacillus*, *Neisseria*, *Staphylococcus,* and *Streptococcus* either increase or decrease in abundance depending on sampling and age. Additionally, the abundance of *Streptococcus danieliae*, *Moraxella osloensis,* and some unclassified *Streptococcus* was affected by the sampling method. While swab and tissue biopsies both identified the common colonizers of oral microbiota, cotton swabbing is a low-cost and practical method, validating the use of the swab as the preferred oral sampling approach.

## 1. Introduction

Gastrointestinal microbiota has a strong relation with metabolism and the modulation of individual health [1]. For the last decades, microbiota research has especially focused on stool and colon, the latter being considered the niche with the highest bacterial density with estimates of 3.9 × 10^13^ bacterial cells [2]. The human oral cavity is colonized by more than 700 bacterial species, making it the second most diverse site after the colon [3]. The combination of the mucosal shredding surfaces from the tongue, internal cheeks, and the hard tooth surfaces creates different environments for the adherence of bacteria in the mouth [4]. Subsequently, the biofilms in the oral cavity vary in bacterial composition and abundance, making the mouth a polymicrobial niche [5]. Several aspects can influence the composition of the oral microbiota, as the mouth is in direct contact with the exterior. Additionally, internal physiological factors, such as aging, could also play a role in the microecological structure [6]. During aging, the salivary flow and composition changes, the cellular exchange modifies, and the loss of dental pieces is frequent [7,8], being those aspects that could also influence the adherence and growth of different bacterial species.

For human samples, the Human Microbiome Project has specifications on how to collect oral samples in the different environments of the human mouth [9,10]. However, mouth samples from mouse models are broadly used to understand the behavior of the microbiota and to associate with diseases and the effect of dietary treatments in the gut [11,12]. In mouse models, the protocols for sampling the oral microbiota are not standardized and the reduced space in the oral cavity is difficult for the sampling. Several forms of sample collection have been used in mouse models including cotton swabbing, extraction of dental pieces, and tissue biopsy [13,14]. Cotton swabbing is an inexpensive and practical method for the collection of oral microbiota without the need to cut tissues, use sedatives, or sacrifice the animal; however, it is not clear if it is the adequate method to characterize the oral microbiota. The aim of this study was to determine whether there is a difference in the oral microbiota based on the sampling approaches and if the age of mice influences the bacterial composition in the oral cavity.

## 2. Materials and Methods

Male C57BL/6 mice were bred in the Central Experimental Animal Husbandry (ZET) at the University Hospital Jena, Jena, Germany. The animals were housed in groups of 8 mice in standard 820 cm^2^ cages (Type III 1290D Tecniplast, Varese, Italy), according to EU guidelines (100 cm^2^ / mouse) with access to food and water ad libitum. Mice were checked daily and the cage was changed weekly, in the case of necessity, the cage was changed more often according to dirtiness to avoid cyclical bias [15]. The mice were fed a standard diet with pellets from ssniff (V1534-300, 10 mm pellets, 9 kj% fat, 24 kj% protein, 67 kj% carbohydrates, for a detailed composition see: http://www.ssniff.de/documents/01-1%20%20DE%20RM%20&%20low%20phyt.pdf, ssniff Spezialdiäten GmbH, Soest, Germany). Resin-free granules of cottonwood were used as bedding (LASbedding PG3, B.LBPG3.10A, granules 3–6 mm, LASvendi GmbH, Soest, Germany). The mice were subjected to strict hygienic controls according to official standards and veterinary regulations. The hygiene status according Federation of European Laboratory Animal Science Associations (FELASA) was recorded by regular deductions and documented by means of health certificates by the specialized staff of ZET. This study was carried out in strict accordance with the recommendations of the European Commission on the protection of animals used for scientific purposes, and all procedures were performed according to the ARRIVE guidelines [16].

Samples from three to seven healthy C57BL/6 male mice aged between 2 and 15 months, respectively, were collected by swabbing the oral cavity and removal of tissue from the mouth immediately after sacrifice by cervical dislocation [number of approval: twz25-2017, date of approval: 06 April 2017, administration: animal protection at University Hospital Jena, Indication of the killing of vertebrates for scientific purposes according to §4 (3) Animal Welfare Germany (from 18 July 2016)]. Samples were immediately snap frozen in liquid nitrogen and stored at −80 °C. The total DNA was extracted following the Trizol protocol (Trizol, Sigma Aldrich, Darmstadt, Germany) with a preliminary step of bead beating (30 s, 5.5 m/s) in a FastPrep instrument (MP Biomedicals). DNA extracts were stored at −20 °C.

Library preparation was performed by targeting the V1-V2 region of the 16S rRNA gene according to the Illumina protocol described by Kaewtapee et al. (2017) [17] with a pre-PCR that amplified the region of interest. The master mix was prepared using PrimeSTAR^®^ HS DNA Polymerase kit (TaKaRa, Beijing, China), 2 µl of DNA template, 0.2 μM of primer, and 0.5 U Taq primer star HS DNA (TaKaRa, China) in a 25 µl volume for the pre and second PCR and 50 µl for the third.

PCR reactions were held at an initial denaturation temperature of 95 °C for 3 min, followed by 10 cycles for the pre and second PCR and 20 cycles for the third PCR following the protocol: 98 °C denaturation for 10 s, annealing of 55 °C for 10 s, and an extension of 72 °C for 45 s with a final extension of 72 °C for 2 min. Libraries were standardized and purified using SequalPrep Normalization Kit (Invitrogen Inc., Carlsbad, CA, USA) and sequenced using 250 bp paired-end sequencing chemistry on an Illumina MiSeq platform.

Sequencing reads were processed using MOTHUR as indicated on the MiSeq SOP [18]. Quality filtering was performed and chimeras were identified and removed by UCHIME. Sequences were aligned against the database Silva version 132. Sequences from chloroplasts, mitochondria, archaea, and eukaryotes were deleted before the OTU clustering at 97% identity. The cut-off for bacterial taxonomy was followed, as described by Yarza et al. (2014) [19]. Data were submitted to the European Nucleotide Archive under the accession number PRJEB32736. Sample reads were standardized by total and a comparison between samples was made by creating a sample-similarity matrix using the Bray-Curtis similarity coefficient (Primer 7) [20]. The differences between the microbial community structure associated with the sampling method and age were identified using Permutational Analysis of Variance (PERMANOVA). For the visual hierarchical clustering and ordination of the community structures, a two-dimensional Principal Coordinate Analysis (PCoA) was created. To assess bacterial diversity, Shannon’s Diversity was calculated. The similarity percentage analysis (SIMPER) was used to identify the OTUs contributing to the observed differences in the oral microbiota sampling. The differences in the abundance of specific OTUs between the treatments were determined with the unpaired Welch’s t-test with a cut-off *p*-value < 0.05. Figures were produced using the web-based tool MicrobiomeAnalyst [21]. Further statistical analyses were calculated using R and SPSS.

## 3. Results

### 3.1. General Microbial Composition Analysis from the Oral Bacterial Community at Different Ages Using Cotton Swab and Tissue Biopsies

**Figure 1:**
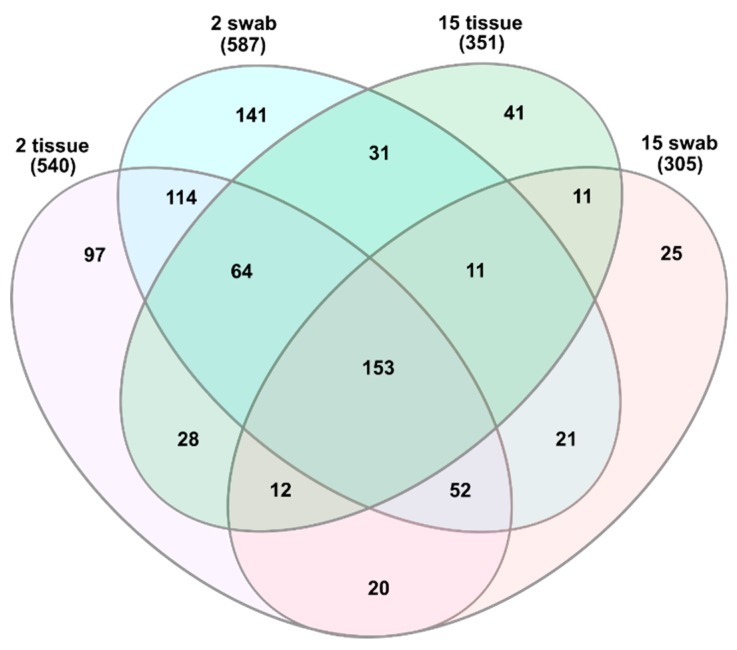
Venn diagram showing the shared and unshared Operational Taxonomic Units between all groups.

A total of 26,366 ± 13,296 sequencing reads were obtained per sample after quality filtering. Reads were clustered into 821 Operational Taxonomic Units (OTU) that were assigned to 210 genera with 20 genera detected on average abundances higher than 0.5%. The core microbiota comprised of 153 OTUs, which were shared between all samples regardless of the type of sampling or age of the animals (Figure 1). Swab samples obtained from 2 months old mice had more unique OTUs than tissue biopsies (141 vs. 97) corresponding to a sum of 28% of the total OTUs. These numbers were lower in 15 months old animals kept in the same facility, as 25 unshared OTUs were detected in swab-samples and 41 in tissue biopsies, which together correspond to a sum of 8% of the total OTUs.

**Figure 2:**
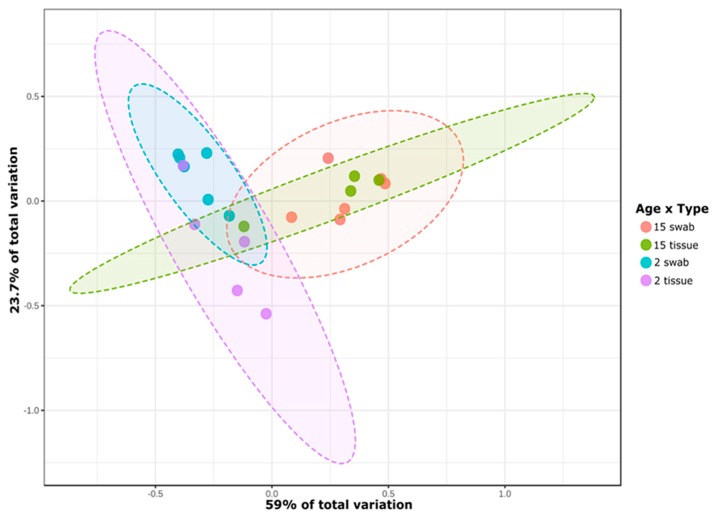
PCoA plot based in Bray Curtis distance matrix. Each point represents one sample from the different groups. Sample spread was statistically different based on Permutational Analysis of Variance (PERMANOVA) analysis (*p*-value < 0.05). Age is represented in months.

The two-dimensional PCoA revealed a clustering in the community similarity structure among the different methods of sampling and age groups (Figure 2). Statistical differences were tested using Permutational Analysis of Variance (PERMANOVA) and differences in the microbiota composition were observed between the two age groups (*p*-value 0.001), sampling methods (oral swab and tissue biopsy) (*p*-value 0.001), and the interaction of both factors (*p*-value 0.01).

Swab sampling showed the highest average similarity between samples when compared to tissue biopsies. In young mice swab samples were 66% similar, while similarity among 15 months old mice was 65%. Tissue average similarities were 39% in 2 months old mice and 46% in 15 months old mice, respectively.

Shannon’s diversity index [22] neither showed statistical significance regarding sampling methods nor for the interaction between both factors. In contrast, statistical significant differences were observed between both young and old mice (*p*-value 0.00), independently of the sampling type with the diversity of microorganisms being higher in 15 months old mice than in young animals (Figure S1).

### 3.2. Taxonomical Bacterial Variation Related to Sampling Approaches and Aging

**Figure 3:**
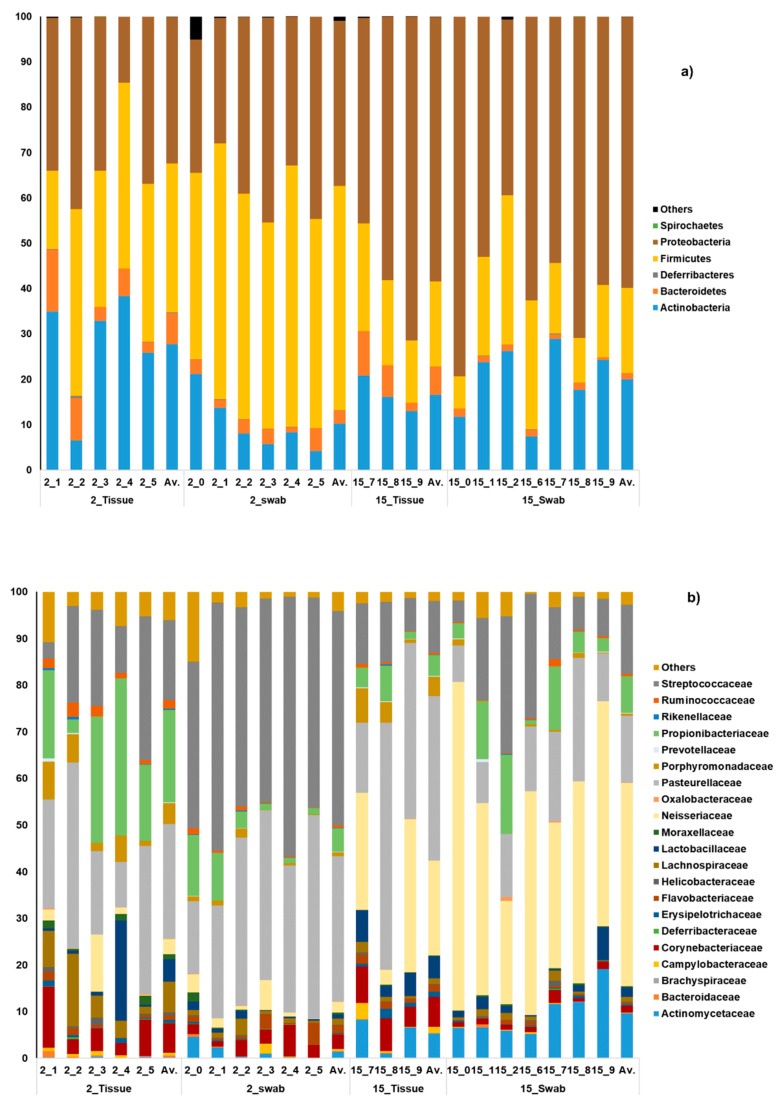
Bacterial community composition at phyla (**a**) and family (**b**) level. Age is represented in months (ex. 2_tissue: Tissue biopsies of 2 months old mice). (**a**) The average relative abundance of each phyla (**a**) and family (**b**) detected in each sample of cotton swab and tissue biopsies from each age group are shown alongside each other. The average (Av.) of each set of samples is shown in the last column of each group.

Firmicutes was the principal phylum detected in the 2 months old mice, while in 15 months mice, Proteobacteria was predominant. Swab samples of 2 month old mice showed a higher abundance of Firmicutes compared to the tissue biopsies obtained from the same mouse (49% and 33%, respectively) (*p*-value 0.02). At 15 months of age, none of the bacterial phyla showed statistical differences between both types of sampling. Phyla pattern was similar between swabs and tissues (Figure 3a).

Actinobacteria, the third most abundant phylum present in the samples, was detected in higher abundance in tissue biopsies obtained from 2 months old mice (28%) when compared to the swab samples obtained from the same animals (10%) (*p*-value 0.03) (Figure 3a).

At the family level, statistical differences were detected among the swab and tissue biopsy samples obtained from 2 months old mice for the families Porphyromonadaceae, Propionibacteriaceae, Ruminococcaceae, and Streptococcaceae (*p*-value ≤ 0.05). In samples obtained from 15 months old animals, the abundance of the families Corynebacteriaceae and Flavobacteriaceae was significantly different (*p*-value ≤ 0.05) (Figure 3b).

When comparing swab samples of young and old mice differences in the family Actinomycetaceae (*p*-value ≤ 0.05) were found, while the abundance of Neisseriaceae, Pasteurellaceae, and Streptococcaceae (*p*-value ≤ 0.05) differed in biopsies obtained from young and old mice (Figure 3b).

**Figure 4:**
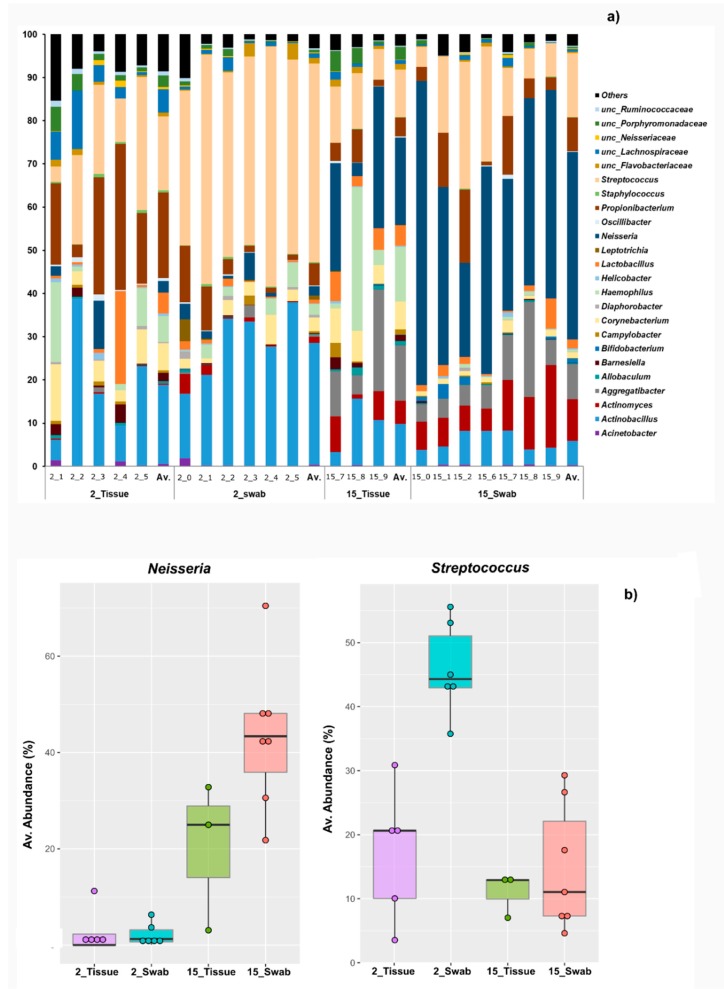
Bacterial community composition at genus level. Age is represented with months (ex. 2_tissue: Tissue biopsies of 2 months old mice). (**a**) The average relative abundance of each genus detected in each sample of cotton swab and tissue biopsies from each age group are shown alongside each other. The average (Av.) of each set of samples is shown in the last column of each group. (**b**) Box plots representing the average abundance of the genera *Neisseria* and *Streptococcus,* each dot represents one sample. For the means, standard deviation of the mean (SEM), variance, and 95% confidence intervals of each genus go to Table S1 (Supplementary Materials).

At genus level, *Streptococcus* was one of the most abundant groups of microorganisms in the oral samples of young mice, however, in samples of 15 months old mice, *Neisseria* was detected in higher abundance than *Streptococcus* (Figure 4). When comparing biopsies and swab samples obtained from 2 months old mice, we observed variances in the relative abundance of *Propionibacterium*, *Streptococcus*, *Clostridium XlVa,* and an unclassified member of *Ruminococcaceae* (*p*-value ≤ 0.05). In contrast, in samples derived from 15 month old animals, *Corynebacterium* (*p*-value ≤ 0.05) was the only genus that showed statistical differences being more abundant in the swabs than in the biopsies (Figure 4a).

The comparison of the cotton swab microbial community from the two different mouse age groups showed a statistical difference in the common mouth colonizers *Actinobacillus, Actinomyces, Aggregatibacter, Neisseria, Staphylococcus, Streptococcus,* and an unclassified member of *Clostridiales* (*p*-value ≤ 0.05) (Figure 4). The microbial community determined in tissue biopsies samples revealed more stability across different ages; however, *Propionibacterium, Streptococcus,* and an unclassified member of *Ruminococcaceae* revealed statistical significance between the sampling groups at 2 months of age (*p*-value ≤ 0.05) (Table 1). At 15 months of age, *Neisseria* and an unclassified member of the Porphyromonadaceae family showed statistically different abundances.

**Figure 5:**
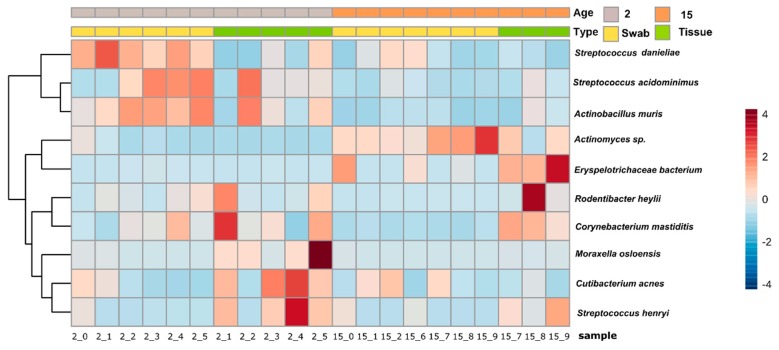
Hierarchical clustering heatmap showing the variation of taxonomic abundances of identified species with statistical differences between the sampling type and age. The color range is represented as a Z-score representing the number of standard deviations above or below the mean.

At the species level, several bacteria showing statistical difference between cotton swab and tissue biopsies could not yet be assigned to a specific species and therefore remained named as unclassified bacteria. In 2 month old mice, *Cutibacterium acnes* was detected in higher abundance in the tissue biopsies (19.7%) in comparison to swab samples (5%), while *Streptococcus danieliae* was more abundant in swab (34%) than in the tissue samples (8.8%) (*p*-value ≤ 0.05) (Figure 5.) Both of these species contributed to the dissimilarity observed between the two age groups. In 2 month old mice, several low abundant unclassified bacteria belonging to *Streptococcus* had statistical differences compared to the older 15 month old mice. Likewise, other low abundant species followed the same pattern: *Erysipelotrichaceae bacterium*, *Moraxella osloensis*, and *Streptococcus henryi* (*p*-value ≤ 0.05). 

## 4. Discussion

This study demonstrated that different oral sampling approaches influence the resultant composition of the microbiota present in the oral cavity. In the present study, we showed that oral swabs and tissue biopsies differed regarding their microbial ecology. Our findings point out that aging impacts the oral microbiota by modifying the composition and diversity of the oral niche. This research is subjected to the sample size limitations, however we could identify statistical differences in the microbial communities for different sampling methodologies that were possibly related to the aggregated microbial community of the diverse mice oral microenvironments. Whereas the oral swab could collect bacteria present in higher abundances in the saliva, tongue, and shedding tissue surfaces, biopsies could be a better screening of the bacteria attached to the oral mucosal areas and thus in closer contact with the host. The overall diversity of the oral cavity samples from 15 months old mice was higher than the diversity of younger animals. Generally, higher microbial diversity is related to a good health status [23]. In line with our findings in the younger mice, in humans, the gut bacterial diversity of newborns remains low and increases with age exponentially during the first three years and continues to increase until adulthood at a lower rate, yet in old age individuals the microbial diversity tends to decrease [24,25]. In this study, we also hypothesized that several intraoral conditions such as salivary flow, lip, or cheek movement and chewing forces could have an impact in the microbial ecology composition [26]. Such factors are influenced by the changes occurring during aging, thus inducing shifts in the bacterial community as at 15 months of age. C57BL/6 mice can be considered between middle and old age at 15 months of life as at this age, this strain has already develop some senescent changes [27]. However, in other mice strains at 15 months, they are considered old because of the behavioral and physical changes that can be detected [28,29].

The higher abundance of *Streptococcus* found in the swab samples obtained from 2 month old mice could result from adhesins present in the *Streptococci* bacteria, which interact with salivary agglutinins that bind bacteria to saliva-coated surfaces and influence the formation of biofilms [30]. The establishment of polymicrobial biofilms starts in the oral cavity from the first days of life [31] when different bacteria colonize mucosal tissue, teeth, tongue, and anaerobic pockets. In human samples, these biofilms have shown to be niche dependent, heterogeneous, and diverge across the ages [32]. The biofilm formation depends on several factors such as co-adhesion, pH, oxygen, and nutrients. Most of the significant bacterial differences between the biopsy and the swab samples were common oral colonizers, implying that the biofilm composition diverges in the oral cavity niches of this mouse model. We consider that the differences observed between the oral sampling approaches of the microbiota could be associated with the attached bacteria in the tissue biopsies.

Similar to findings in humans, we revealed high inter-individual differences in several usual mouth colonizers, such as Streptococcus and Neisseria [33], the last one identified as part of the core microbiota of the dental plaque and saliva [32]. Those differences were not only inter-individual, but also between the sampling types. One of the advantages of mice models is that lifestyle, diet, and personal environment are factors without weight in the results when compared to human studies. Therefore, we can conclude that age and sampling procedures are elements that influence the identification and quantification of the oral bacterial communities in mice models and those are factors that could also impact the human oral microbial niches. As we found clear differences related to the sampling approaches and the age effect on oral microbiota, further research is required to understand how the bacterial ecology in the mouth fluctuates through the aging process and to establish a protocol that can be comparable between research groups.

## 5. Conclusions

This study suggests that the sampling method is a factor to consider when determining bacterial abundances and diversity in the oral cavity. Indeed, we showed that in oral swab samples, bacterial abundances and diversity differ from those found in biopsies of the oral tissue. As the oral cavity in mice and human is colonized with biofilms, oral swabs could depict the overall composition of the oral niches, while the tissue biopsies could be representative of the soft tissue. Nevertheless, as a similar bacterial composition was found in the cotton swab and the biopsies, we consider it as adequate to use an oral cotton swab for sampling in order to assess the microbiota of the oral cavity as it is a cost-effective and practical approach to collect bacteria from the different oral microbial microenvironments in mice models.

## Figures and Tables

**Table 1:** Genera showing differences between sampling types.

Phyla	Genera	Cotton Swab				Tissue Biopsy				
		2 Months		15 Months		2 Months		15 Months		*p*-Value
		Mean	95% CI	Mean	95% CI	Mean	95% CI	Mean	95% CI	
Actinobacteria	*Propionibacterium*	5.1 *	−0.3–34.2	7.8	1.9–13.7	19.8 *	5.2–34.3	4.4	1.9–13.7	0.05
Firmicutes	*Streptococcus*	45.9 *	38.3–53.5	14.8	5.6–23.9	17.1 *	4–30	10.9	5.6–23.9	0.00
Firmicutes	unc_*Ruminococcaceae*	0.3 *	0.4–1.4	0.2	0.1–0.3	0.9 *	0–0–6	0.2	0.1–0.4	0.02
Proteobacteria	*Neisseria*	2.1	−0.3–4–6	43.3 *	29.1–57.5	2.7	−3.3–8.7	20.2 *	29.1–57.5	0.03
Bacteroidetes	unc_*Porphyromonadaceae*	0.6	0.03–1.3	0.3 *	−0.03–0.7	2.6	0.1–5.2	2.9 *	−0.03–0.7	0.05

* Compared samples with statistical difference.

## Supplementary Materials

The following are available online at https://www.mdpi.com/2076-2607/7/9/283/s1, Figure S1: Alpha diversity calculated by Shannon’s. Table S1. Descriptive statistics from the bacterial genera present in higher abundances in the mice oral samples, at 2 months of age (A) and 15 months of age (B).
