# Supplementary material for: Changes in Oral Microbial Ecology of C57BL/6 Mice at Different Ages Associated with Sampling Methodology
Source: Microorganisms. 2019 Aug 22;7(9):283. doi: 10.3390/microorganisms7090283 (PMC6780121; doi:10.3390/microorganisms7090283)
Supplement: Supplementary file 1 [file microorganisms-07-00283-s001.pdf]

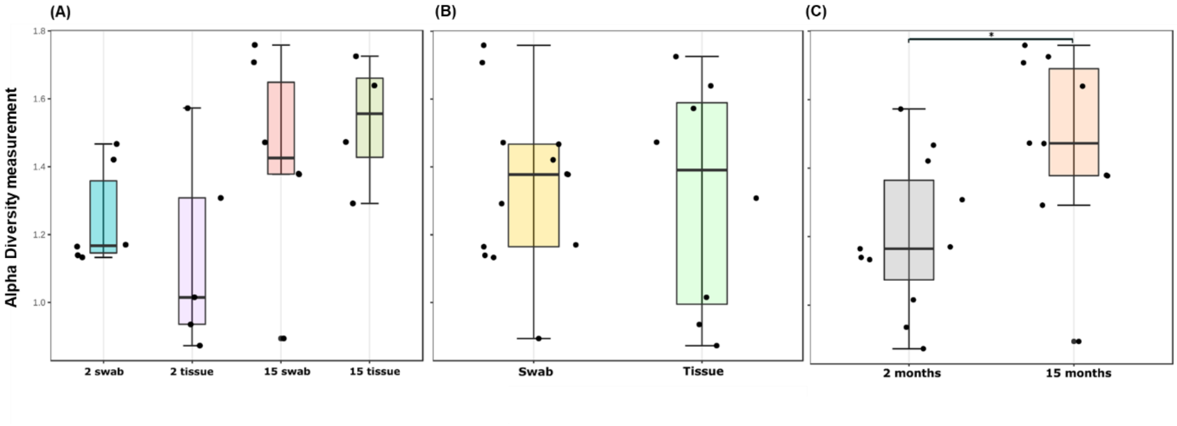

Figure S1. Alpha diversity calculated by Shannon's. Comparison between all groups (A), sampling methodologies (B) and age (C). Alpha diversity compared by age was statistically different (p-value >0.001), represented by (*). Numbers before the sample type refers to the age in months.

(A)

	2_tissue n=5					2_swab n=6				
				95% confidence intervals					95% confidence intervals	
	Mean	Std. Deviation of mean	Variance	Lower Bound	Upper Bound	Mean	Std. Deviation of mean	Variance	Lower Bound	Upper Bound
**Acinetobacter**	0.56	0.29	0.43	-0.25	1.37	0.38	0.30	0.53	-0.38	1.14
**Actinobacillus**	18.34	6.03	182.03	1.59	35.09	28.20	3.58	76.93	18.99	37.40
**Actinomyces**	0.18	0.07	0.03	-0.03	0.39	1.41	0.71	3.06	-0.43	3.24
**Aggregatibacter**	0.23	0.21	0.23	-0.36	0.82	0.55	0.45	1.23	-0.36	0.82
**Allobaculum**	0.38	0.14	0.10	-0.01	0.78	0.11	0.06	0.02	-0.04	0.25
**Barnesiella**	1.89	0.74	2.77	-0.18	3.95	0.15	0.03	0.01	0.07	0.22
**Bifidobacterium**	0.00	0.00	0.00	-0.01	0.02	0.05	0.05	0.02	-0.08	0.19
**Campylobacter**	0.59	0.16	0.13	0.16	1.03	0.40	0.35	0.75	-0.50	1.31
**Corynebacterium**	6.30	1.92	18.50	0.96	11.64	3.23	0.79	3.78	1.19	5.27
**Diaphorobacter**	0.32	0.14	0.10	-0.07	0.72	0.58	0.28	0.49	-0.07	0.72
**Haemophilus**	5.97	3.50	61.22	-3.75	15.68	2.47	0.90	4.85	0.16	4.78
**Helicobacter**	0.63	0.27	0.36	-0.12	1.38	0.15	0.04	0.01	0.05	0.25
**Lactobacillus**	4.80	4.19	87.64	-6.82	16.42	0.87	0.30	0.56	0.08	1.65
**Leptotrichia**	0.00	0.00	0.00	0.00	0.00	0.96	0.83	4.10	-1.16	3.09
**Neisseria**	2.70	2.17	23.63	-3.34	8.73	2.19	0.97	5.65	-0.30	4.68
**Oscillibacter**	0.70	0.25	0.31	0.01	1.39	0.17	0.05	0.01	0.30	5.13
**Propionibacterium**	19.80	5.23	136.64	5.29	34.32	5.13	2.12	27.02	-0.32	10.59
**Staphylococcus**	0.47	0.13	0.08	0.12	0.82	0.30	0.09	0.05	0.08	0.53
**Streptococcus**	17.15	4.73	112.07	4.00	30.29	45.95	2.97	52.87	4.00	30.29
**unc_Flavobacteriaceae**	0.81	0.29	0.42	0.00	1.61	1.28	0.68	2.80	-0.47	3.04
**unc_Lachnospiraceae**	5.45	2.22	24.61	-0.71	11.61	1.10	0.43	1.11	0.00	2.21
**unc_Neisseriaceae**	0.56	0.29	0.43	-0.25	1.37	0.15	0.05	0.02	0.01	0.28
**unc_Porphyromonadaceae**	2.65	0.92	4.23	0.10	5.21	0.66	0.25	0.36	0.03	1.30
**unc_Ruminococcaceae**	0.89	0.17	0.15	0.41	1.38	0.28	0.12	0.08	-0.01	0.58

(B)

	15_tissue n=3					15_swab n=7				
				**95% confidence intervals**					**95% confidence intervals**	
	**Mean**	**Std. Deviation of mean**	**Variance**	**Lower Bound**	**Upper Bound**	**Mean**	**Std. Deviation of mean**	**Variance**	**Lower Bound**	**Upper Bound**
**Acinetobacter**	0.14	0.06	0.01	-0.11	0.39	0.25	0.05	0.02	0.12	0.37
**Actinobacillus**	9.73	3.55	37.78	-5.54	25.00	5.64	0.84	4.93	3.58	7.69
**Actinomyces**	5.31	2.21	14.69	-4.21	14.83	9.61	1.91	25.48	4.94	14.28
**Aggregatibacter**	12.87	5.66	95.94	-11.46	37.20	8.17	2.46	42.24	2.16	14.18
**Allobaculum**	1.01	0.42	0.52	-0.79	2.80	0.16	0.05	0.02	0.03	0.30
**Barnesiella**	1.31	0.76	1.71	-1.94	4.56	0.14	0.06	0.03	-0.01	0.29
**Bifidobacterium**	0.08	0.07	0.02	-0.24	0.39	0.98	0.46	1.45	-0.14	2.09
**Campylobacter**	1.27	1.05	3.34	-3.27	5.81	0.14	0.05	0.02	0.01	0.27
**Corynebacterium**	6.43	1.08	3.51	1.78	11.09	1.35	0.27	0.50	0.69	2.00
**Diaphorobacter**	0.01	0.01	0.00	-0.02	0.04	0.14	0.12	0.10	-0.15	0.43
**Haemophilus**	12.66	10.31	318.80	-31.69	57.01	0.46	0.13	0.12	0.13	0.78
**Helicobacter**	0.23	0.15	0.07	-0.41	0.86	0.27	0.15	0.16	-0.09	0.64
**Lactobacillus**	4.71	1.28	4.95	-0.81	10.24	2.07	0.88	5.40	-0.08	4.22
**Leptotrichia**	0.08	0.08	0.02	-0.25	0.40	0.00	0.00	0.00	0.00	0.00
**Neisseria**	20.28	8.90	237.37	-18.00	58.55	43.37	5.80	235.44	29.18	57.56
**Oscillibacter**	0.22	0.15	0.07	-0.43	0.88	0.18	0.14	0.13	-0.16	0.51
**Propionibacterium**	4.45	1.78	9.53	-3.22	12.12	7.83	2.41	40.71	1.93	13.73
**Staphylococcus**	0.07	0.03	0.00	-0.06	0.20	0.08	0.04	0.01	-0.03	0.18
**Streptococcus**	10.99	1.98	11.77	2.47	19.51	14.82	3.74	98.07	5.66	23.98
**unc_Flavobacteriaceae**	1.27	0.24	0.17	0.25	2.29	0.33	0.11	0.08	0.06	0.59
**unc_Lachnospiraceae**	0.97	0.41	0.51	-0.80	2.74	0.65	0.27	0.51	0.00	1.31
**unc_Neisseriaceae**	0.08	0.05	0.01	-0.12	0.27	0.15	0.10	0.07	-0.09	0.40
**unc_Porphyromonadaceae**	2.98	1.21	4.38	-2.22	8.18	0.33	0.15	0.15	-0.03	0.69
**unc_Ruminococcaceae**	0.17	0.04	0.00	0.01	0.33	0.24	0.05	0.02	0.11	0.37

Table S1. Descriptive statistics from the bacterial genera present in higher abundances in the mice oral samples, at 2 months of age (A) and 15 months of age (B).
